# Inverse Association between Serum Bilirubin Levels and Arterial Stiffness in Korean Women with Type 2 Diabetes

**DOI:** 10.1371/journal.pone.0109251

**Published:** 2014-10-09

**Authors:** Eun Sook Kim, Eun young Mo, Sung Dae Moon, Je Ho Han

**Affiliations:** 1 Division of Endocrinology and Metabolism, Department of Internal Medicine, College of Medicine, The Catholic University of Korea, Seoul, Korea; 2 The Catholic University of Korea Incheon St. Mary's hospital, Incheon, Korea; University of Milan, Italy

## Abstract

**Background:**

Considerable evidence suggests that bilirubin is a potent physiologic antioxidant that may provide important protection against cardiovascular disease (CVD) and inflammation. We investigated the relationship between serum total bilirubin (TB) levels and arterial stiffness, measured by the brachial-ankle pulse wave velocity (baPWV), in patients with type 2 diabetes.

**Methods:**

We conducted a cross-sectional analysis of 1,711 subjects with type 2 diabetes (807 men and 904 women; mean age, 57.1 years). The subjects were stratified based on gender-specific tertiles of TB values, and a high baPWV was defined as greater than 1,745 cm/s ( >75th percentile).

**Results:**

The serum TB concentration was negatively correlated with the duration of diabetes, HbA1c, the 10-year Framingham risk score, and baPWV and was positively correlated with high-density lipoprotein cholesterol and the eGFR in both genders. Inverse association between TB categories and unadjusted prevalence of high PWV was only observed in women. After adjusting for confounding factors, the TB levels were inversely associated with a greater risk of a high baPWV, both as a continuous variable [a 1-SD difference; odds ratio (OR), 0.70; 95% confidence interval (CI), 0.54–0.90; *P* = 0.005] and when categorized in tertiles (the highest vs. the lowest tertile; OR, 0.49; 95% CI, 0.28–0.85; *P* = 0.011) in women but not in men. The relationship remained significant even after adjusting for retinopathy and nephropathy.

**Conclusions:**

Low TB levels were significantly associated with arterial stiffness in Korean women with type 2 diabetes. Our data suggested that bilirubin may protect against macrovascular disease in diabetic women.

## Introduction

Diabetes mellitus has become a major health issue worldwide. It is estimated that approximately 285 million people worldwide had diabetes in 2010, and this estimate is expected to reach 438 million by the year 2030 [1]. Patients with diabetes are at a higher risk of chronic complications that reduce the quality of life, pose a heavy economic burden on the health care system, and increase the mortality rate. Cardiovascular disease (CVD) is the leading cause of death in patients with type 2 diabetes, with a two- to four-fold increase in incidence compared with patients without diabetes [2]. Current guidelines recommend a multifactorial intervention to control glucose, blood, and lipid levels to provide good diabetes care, but the majority of patients fail to meet these therapeutic target goals [3]. Moreover, intensive glycemic control in randomized controlled trials has not demonstrated a significant decrease in CVD outcomes [4]. Thus, there is ardent interest in identifying novel biomarkers to refine the risk stratification for CVD.

Bilirubin is the end product of heme catabolism and has antioxidant and anti-inflammatory properties [5]. Increasing evidence has demonstrated that mild to moderate increases in the serum bilirubin concentration, even within the normal range, are associated with a reduced risk of CVD, regardless of alcohol drinking or liver damage [6]. Clinical studies have consistently reported inverse associations between serum bilirubin levels and hypertension, obesity, dyslipidemia, metabolic syndrome, and diabetes, all of which raise the risk of CVD. Furthermore, longitudinal studies have revealed that low serum bilirubin levels were a significant predictor of the incidence of vascular diseases, including coronary artery disease, stroke, and peripheral artery disease limb amputation, and mortality, above and beyond conventional risk factors [7]–[10]. However, the association between serum bilirubin levels and CVD in patients with type 2 diabetes has not been established.

Arterial stiffness has been shown to be a principal indicator of the development and progression of cardiovascular disease related to arteriosclerosis [11], [12]. Increased arterial stiffness has been proposed to be an important mechanism for the diabetes-related increase in CV risk, the development of CVD, and mortality [13]. The recently introduced brachial-arterial pulse wave velocity (baPWV) is a reliable and easily accessible measurement of arterial stiffness that is comparable to the carotid-femoral PWV, the golden standard method for assessing arterial stiffness, and is therefore suitable for large-scale evaluations.

This study investigated the association between serum TB concentration and arterial stiffness, measured by baPWV, in patients with type 2 diabetes.

## Methods

### Subjects

We retrospectively recruited subjects with type 2 diabetes who were older than 30 years of age and who visited Incheon St. Mary's Hospital between August 2011 and November 2013 for the purpose of glucose control. Patients with a medical history of chronic hepatic disease, abnormal hepatic function (defined as aspartate aminotransferase (AST) >100 IU/L or alanine aminotransferase (ALT) >100 IU/L), an ankle-brachial pressure index <0.9 or >1.3, severe illness such as systemic inflammatory disease, or a progressive malignancy were excluded, as were those taking warfarin or corticosteroids. A total of 1,711 patients were included in the final analysis.

### Ethics statement

The Institutional Review Board of the Clinical Research Coordinating Center at Incheon St. Mary's Hospital approved the study protocol.

### Ethics statement

This study was approved by the institutional review board of Catholic University Incheon St. Mary's Hospital, Incheon, Korea. Due to the retrospective nature of this study presenting no more than minimal risk of harm to participants, the institutional review board waived the requirement to obtain informed consent.

### Clinical and biochemical assessment

Demographic and clinical data were verified by reviewing the electronic medical records. Body mass index (BMI) was calculated by dividing the patients' weight in kilograms by their height in meters squared. Blood pressure was measured after 5 min of rest, and hypertension was defined as a systolic BP ≥140 mmHg, a diastolic BP ≥90 mmHg, or the use of antihypertension medication. Hyperlipidemia was defined as a triglyceride (TG) concentration ≥150 mg/dL, an LDL concentration ≥100 mg/dL, and/or the use of a cholesterol-lowering medication. Cardiovascular disease (CVD) was defined as previous myocardial infarction, coronary revascularization, or stroke. After overnight fasting, venous blood was taken for laboratory measurements. Hemoglobin A1c (HbA1c) was measured by high-performance liquid chromatography using a Variant II Turbo system (Bio-Rad Laboratories, Hercules, CA). Routine biochemical measurements were performed on each blood sample using an AU5400 automated chemistry analyzer (Beckman Coulter, Fullerton, CA, USA). Total cholesterol and triglycerides were measured using a standard enzymatic method, and high-density lipoprotein (HDL) cholesterol was measured using an enzymatic colorimetric method. LDL cholesterol was indirectly measured using the Friedewald formula in participants with serum triglyceride concentrations below 400 mg/mL. Serum aspartate aminotransferase (AST) and alanine aminotransferase (ALT) levels were measured using a cokinetic UV method based on recommendations by the International Federation of Clinical Chemistry (IFCC). The serum creatinine concentration was measured using a modified kinetic Jaffe method. Total bilirubin was measured by the 2,5-dichlorophenyldiazonium (DPD) method. The albumin-creatinine ratio (ACR) was calculated in spot urine samples, which were categorized into three groups: normoalbuminuria (<30.0 mg/g), microalbuminuria (30.0–299.9 mg/g), or macroalbuminuria (≥300.0 mg/g). Nephropathy was defined as the presence of micro-or macroalbuminuria. The glomerular filtration rate (GFR) was estimated using the Modification of Diet in Renal Disease (MDRD) study equation [14]. Diabetic retinopathy (DMR) was assessed and classified as no DMR, nonproliferative DMR (NPDR), or proliferative DMR (PDR); positive retinopathy included NPDR and PDR. The Framingham risk score (FRS) was calculated to estimate the 10-year risk for coronary heart disease using validated algorithms [15].

### Measurement of brachial-ankle pulse wave velocity (baPWV)

The baPWV was measured using an automated PWV/ABI analyzer (VP-1000; Colin Co. Ltd., Komaki, Japan) after the subjects had rested in the supine position for at least 5 min. ECG electrodes were placed on both wrists and both ankles, and BP cuffs were wrapped around both upper arms and both ankles. To measure the baPWV, pulse waves obtained from the brachial and tibial arteries were recorded simultaneously, and the transmission time (ΔTba) was calculated as the time interval between the initial increase in the brachial and ankle waveforms. The path length from the suprasternal notch to the brachium (Lb) and from the suprasternal notch to the ankle (La) was automatically obtained based on the subject's height. The baPWV was calculated using the equation baPWV  =  (La-Lb)/ΔTba (cm/s), and the mean baPWVs for the left and right sides were used for the analysis.

### Statistical analysis

The statistical analyses were performed using SAS software (ver. 9.1; SAS Institute, Cary, NC, USA). The data are presented as the mean ± SD or as a percentage, and a P value <0.05 was considered statistically significant. Comparisons of two continuous variables were performed using Student's t-test, and categorical variables were analyzed using the χ^2^ test to compare the characteristics of the study population. *Pearson* correlation analyses were performed to examine the association between PWV and various parameters. Due to skewed distribution, log-transformed TB and TG were used. A high PWV was defined as the highest quartile of values among the subjects (≥1745 cm/s). Multivariate logistic regression analyses were performed to estimate the odds ratio (OR) and the 95% confidence interval (CI) for high PWV. *P* values of <0.05 were considered statistically significant.

## Results

### Clinical characteristics of the participants

The clinical characteristics are presented in Table 1. The mean age of the participants was 57.1±10.5 years, and 52.8% of the participants were women. The mean duration of type 2 diabetes was 7.6±7.7 years. The prevalence of smokers, drinkers, and past CVD and the DBP, TG, TB, ALT, and 10-year FRS were significantly higher in the men than in the women; however, the women were older and had a longer duration of diabetes, higher SBP, LDL-C levels, and a higher PWV than the men. The prevalence of retinopathy and nephropathy and the HbA1c levels were not different between the genders.

**Table 1:** Clinical characteristics of the subjects.

	Men	Women	*P*
n	807	904	
Age (years)	55.2±10.1	58.8±10.8	<0.001
DM duration (years)	7.0±7.7	8.1±7.8	0.002
Retinopathy (%)^a^	27.2	26.2	0.677
Nephropathy (%)^b, c^	27.2	28.3	0.637
History of CVD (%)	11.5	7.7	0.008
Insulin use (%)	24.0	26.3	0.277
Statin use (%)	32.2	33.0	0.635
ACEi/ARB use (%)	31.9	35.1	0.159
Smoking (%)	38.1	5.1	<0.001
Alcohol (%)	56.6	17.6	<0.001
BMI (kg/m2)	24.6±3.2	25.1±3.7	0.006
HbA1c (%)	8.2±2.2	8.1±2.1	0.487
SBP (mmHg)	129.0±16.3	130.8±18.3	0.032
DBP (mmHg)	79.7±10.1	77.1±10.7	<0.001
TC (mg/dL)	177.1±45.6	180.3±44.4	0.148
TG (mg/dL)	189.6±143.8	162.9±111.4	<0.001
HDL-C (mg/dL)	43.9±10.7	47.6±11.9	<0.001
LDL-C (mg/dL)	96.5±37.5	100.7±37.2	0.030
eGFR (ml/min/1.73 m^2^)^d^	103.1±28.5	103.3±33.1	0.892
TB (mg/dL)	0.82±0.35	0.67±0.26	<0.001
AST (IU/L)	25.9±11.9	26.0±12.3	0.862
ALT (IU/L)	28.9±17.0	26.4±16.5	0.002
10-year FRS (%)	10.5±7.3	3.5±4.1	<0.001
PWV (m/s)	15.3±3.0	16.1±3.6	<0.001

Data are expressed as means ± SD or percentage unless otherwise indicated.

CVD indicates cardiovascular disease; ACEi/ARB, angiotensin converting enzyme inhibitors/angiotensin receptor blockers; BMI, body mass index; HbA1c, hemoglobin A1c; SBP, systolic blood pressure; DBP, diastolic blood pressure; TC, total cholesterol; TG, triglycerides; HDL-C, high-density lipoprotein cholesterol; LDL-C, low-density lipoprotein cholesterol; eGFR, estimated glomerular filtration rate; TB, total bilirubin; AST, aspartate aminotransferase; ALT, alanine aminotransferase; FRS, Framingham Risk score; PWV, pulse wave velocity

a measured in 1292 subjects (613 men and 679 women), ^b^measured in 1419 (677 men and 742 women), ^c^Nephropathy was defined as the presence of micro-or macroalbuminuria,^ d^The eGFR was calculated using the Modification of Diet in Renal Disease study equation

### Correlations between TB concentration and various parameters

Serum TB concentration was negatively correlated with the duration of diabetes, HbA1c, the 10-y FRS, and the PWV and was positively correlated with HDL-C levels, and the eGFR, hepatic enzyme levels in both men and women (Table 2). The serum TB concentration was negatively correlated with age, TC and TG levels in men but did not correlate with BMI, SBP, DBP, or LDL-C in either gender.

**Table 2:** *Pearson* correlation between log-transformed serum total bilirubin concentration and clinical variables.

	Men		Women	
	r	*P*	r	*P*
Age (years)	−0.07	0.036	−0.02	0.523
DM duration (years)	−0.18	<0.001	−0.22	<0.001
BMI (kg/m2)	0.06	0.067	0.01	0.653
HbA1c (%)	−0.12	0.001	−0.11	0.001
SBP (mmHg)	−0.04	0.266	−0.05	0.101
DBP (mmHg)	0.01	0.795	0.02	0.613
TC (mg/dL)	−0.07	0.042	0.01	0.681
TG (mg/dL)*	−0.10	0.006	−0.04	−0.237
HDL-C (mg/dL)	0.14	<0.001	0.09	0.012
LDL-C (mg/dL)	−0.05	0.219	0.01	0.682
eGFR (ml/min/1.73 m^2^)	0.14	<0.001	0.16	<0.001
AST (IU/L)	0.21	<0.001	0.11	0.001
ALT (IU/L)	0.20	<0.001	0.10	0.004
PWV (cm/s) *	−0.10	0.004	−0.13	<0.001
ABI	−0.03	0.464	−0.02	0.358
10-yr FRS (%)	−0.16	<0.001	−0.08	0.019

*Tested by log-transformed.

### Comparison of cardiometabolic risk parameters and PWV based on TB levels

**Figure 1:**
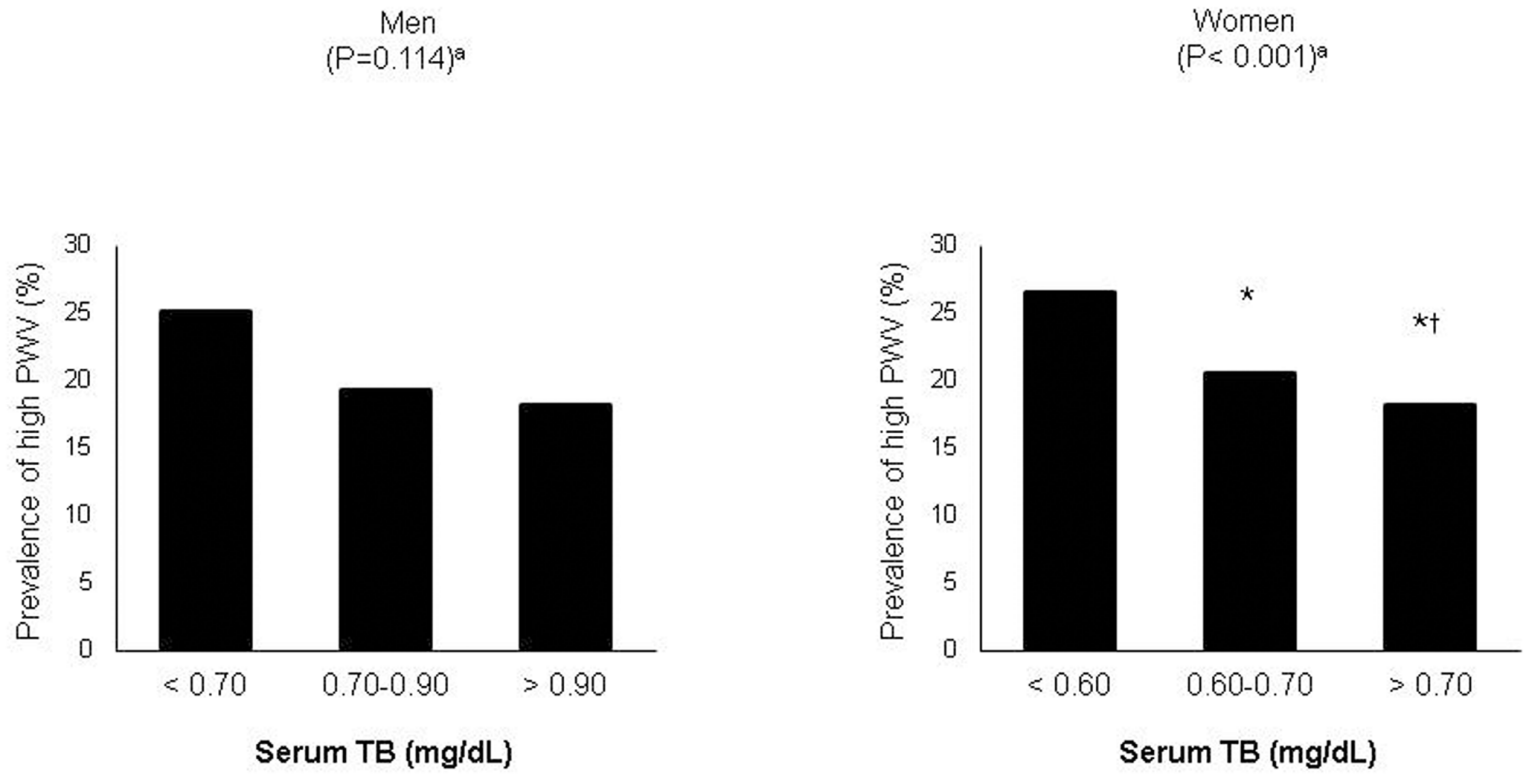
Unadjusted prevalence of a high PWV according to serum TB levels in men and women. ^a^by χ2 test over the three groups; ^*^ *P*<0.05 vs. T1 (TB <0.60 mg/dL), ^#^ *P*<0.05 vs. T2 (TB 0.60–0.70 mg/dL).

The characteristics of the subjects according to the tertiles of serum TB levels are summarized in Table 3. Subjects of both genders in the third tertile had a shorter duration of diabetes, a decreased prevalence of retinopathy and nephropathy, lower HbA1c levels, and PWV whereas a lower 10-y FRS (%) in the second and third tertile was observed in men. When we assessed the association between serum TB and PWV as a categorical variable, we found that inverse association between TB categories and unadjusted prevalence of high PWV only in women (Fig.1).

**Table 3:** Comparison of clinical characteristics according to TB tertiles in men and women.

	Men			Women		
TB (mg/dL)	T1	T2	T3	T1	T2	T3
	(<0.70)	(0.70–0.90)	(>0.90)	(<0.60)	(0.60–0.70)	(>0.70)
n	271	295	241	326	302	276
Age (years)	55.9±10.7	55.0±9.9	54.5±9.6	58.6±11.0	59.3±10.4	58.6±11.0
DM duration (years)	8.4±8.2	6.9±7.7	5.3±6.6^a^ ^,b^	10.1±8.7	7.8±7.5^a^	6.1±6.2^a^ ^,b^
Retinopathy (%)† ^‡^	38.6	24.7	17.4	37.3	18.2	22.2
Nephropathy (%)† ^‡^	36.8	24.6	19.3	36.3	21.5	27.1
History of CVD (%)†	15.5	11.2	7.5	7.4	8.0	8.0
Insulin use (%)† ^‡^	31.7	21.4	18.7	35.3	22.9	19.6
Statin use (%)	34.7	32.9	28.6	31.6	35.4	33.0
ACEi/ARB use (%)	35.8	32.5	26.6	34.1	37.4	33.7
Smoking (%)	42.8	37.4	33.6	7.1	4.3	3.6
Alcohol (%)	52.1	58.4	59.6	15.8	19.9	17.2
BMI (kg/m2)	24.4±3.3	24.6±3.1	24.8±3.2	25.1±3.9	24.8±3.5	25.5±3.7^b^
HbA1c (%)	8.5±2.4	8.2±2.2	7.9±1.9^a^	8.4±2.1	8.1±2.2	7.9±1.9^a^
SBP (mmHg)	131.0±16.6	127.3±16.0^a^	128.8±16.2	131.9±18.8	130.5±17.8	129.8±18.0
DBP (mmHg)	80.1±10.4	79.1±9.9	79.9±10.1	77.2±11.4	76.5±9.8	77.5±10.6
TC (mg/dL)	180.2±48.6	177.8±45.0	172.8±42.6	178.4±44.4	183.0±4.6	179.6±41.7
TG (mg/dL)	204.1±155.7	181.2±125.3	183.6±150.5	173.1±121.8	154.4±119.6	160.5±85.8
HDL-C (mg/dL)	42.1±10.8	44.4±10.2^a^	45.4±11.0^a^	45.7±11.5	49.2±12.7^a^	48.1±10.1
LDL-C (mg/dL)	98.4±37.5	97.5±37.6	93.2±37.3	98.8±37.0	103.3±36.1	99.9±38.5
eGFR (ml/min/1.73 m^2^)	99.1±31.9	103.5±25.2	107.1±27.8^a^	96.3±36.0	108.5±33.7^a^	105.9±27.2^a^
AST (IU/L)	23.3±10.6	25.9±10.8^a^	29.0±13.8^a^ ^,b^	24.8±11.9	25.0±10.6	28.3±13.9^a^ ^,b^
ALT (IU/L)	25.1±15.0	29.1±15.6^a^	33.2±19.8^a^ ^,b^	25.2±16.7	24.8±14.9	29.1±16.5^a^ ^,b^
10-year FRS (%)	12.0±7.7	10.1±7.2^a^	9.2± 6.6^a^ ^,b^	3.8±4.5	3.6±4.1	3.1±3.7
PWV (m/s)	15.8±3.3	15.0±2.7^a^	15.1±2.9^a^	16.6±3.7	16.0±3.5	15.6±3.4^a^

a *P*<0.05 vs. T1 and ^b^ *P*<0.05 vs. T2 (One-way ANOVA and post hoc test).

† *P*<0.05 in men and ^‡^ *P*<0.05 in women (χ^2^ test).

### Association between serum TB levels and the risk of high PWV

In a multivariate analysis adjusted for age, BMI, the duration of diabetes, drinking, and smoking status, a history of CVD, HbA1c, SBP, DBP, ALT, TC, TG, HDL-C, eGFR and the use of insulin, ACEi/ARB, or statins (Table 4; model 2), TB levels were inversely associated with a greater risk of a high PWV, both as a continuous variable [a 1-SD difference; odds ratio (OR), 0.70; 95% confidence interval (CI), 0.54–0.90; P = 0.005] and when categorized in tertiles (the highest vs. the lowest tertile; OR, 0.49; 95% CI, 0.28–0.85; P = 0.011) in women. Further adjusting model 2 for retinopathy and albumin-to-creatinine ratio did not change the results of the analysis. However, there was no association in men between TB levels and the PWV as continuous or categorized variables.

**Table 4:** Odds ratios (95% CI) for high PWV according to TB levels in men and women.

	Men		Women	
	OR (95% CI)	P	OR (95% CI)	P
**Model 1**				
Per SD of TB	1.02 (0.84–1.24)	0.840	0.71 (0.58–0.86)	0.001
By TB tertiles				
T1	1		1	
T2	0.80 (0.52–1.25)	0.465	0.64 (0.43–0.95)	0.640
T3	0.87 (0.54–1.40)	0.878	0.48 (0.32–0.74)	0.009
**Model 2**				
Per SD of TB	1.20 (0.93–1.55)	0.156	0.70 (0.54–0.90)	0.005
By TB tertiles				
T1	1		1	
T2	1.37 (0.77–2.42)	0.288	0.67 (0.40–1.11)	0.122
T3	1.52 (0.80–2.86)	0.200	0.49 (0.28–0.85)	0.011
**Model 3**				
Per SD of TB	1.21 (0.88–1.68)	0.245	0.58 (0.42–0.82)	0.002
By TB tertiles				
T1	1		1	
T2	1.41 (0.67–2.94)	0.364	0.54 (0.28–1.04)	0.065
T3	1.35 (0.59–3.07)	0.473	0.32 (0.16–0.65)	0.002

Model 1: adjusted for age, BMI, duration of diabetes.

Model 2: adjusted for the variables in model 1 and drinking and smoking status, history of CVD, HbA1c, SBP, DBP, ALT, TC, TG, HDL-C, eGFR, use of insulin, ACEi/ARB, and statin.

Model 3, adjusted for the variables in model 2 and retinopathy and albumin-to-creatinine ratio.

## Discussion

In this study, the TB levels were negatively correlated with diabetes duration, HbA1c levels, the eGFR, and the 10-y FRS but were associated with a higher PWV, suggesting a close relationship between TB levels and reduced CVD risk. Moreover, individuals in the lower tertile of TB levels had an increased prevalence of microvascular complications such as diabetic retinopathy and nephropathy as well as previous CVD history. The serum TB concentration was inversely associated with increased baPWV in women with diabetes after adjusting for microvascular complications and conventional cardiovascular risk factors. However, there was no association between TB levels and baPWV in men.

Notably, type 2 diabetes is related to vascular complications, the leading cause of morbidity, disability, and premature death, which could not be explained by clustering the classic risk factors. Epidemiological studies have reported that arterial stiffness is closely correlated with surrogate markers of atherosclerosis and that baPWV, a measure of arterial stiffness, is an independent predictor of adverse cardiovascular events. Arterial stiffness is increased in patients with impaired glucose tolerance and T2DM and is closely associated with diabetic microvascular disease, suggesting that it could be a pathogenic mechanism of hyperglycemia-induced CV risk that is initiated before the onset of diabetes and may provide a potential explanation for the intimate link between microvasculopathy and macrovascular disease. As oxidative stress is a shared mechanism underlying endothelial dysfunction, microangiopathy, and atherosclerosis in hyperglycemia, it is plausible that bilirubin, an innate antioxidant, may exert a protective effect against atherosclerosis. Accordingly, Inoguchi et al. [16] have reported that diabetic patients with Gilbert syndrome (n = 96), the most common hereditary genetic disorder causing hyperbilirubinemia, had a lower prevalence of coronary artery disease, cerebrovascular disease, and microvascular complications than those without Gilbert syndrome (n = 426). However, the data on patients with diabetes, not confined to those with Gilbert syndrome, are sparse and limited by small sample sizes and inconsistent results. Dullaart et al. reported a similar negative association between serum bilirubin levels and carotid IMT in 40 non-diabetic and 80 diabetic subjects [17]. In contrast, Yeh et al. found no association between bilirubin levels and vascular reactivity in the macro- and microcirculation of 37 patients with type 1 diabetes and 213 patients with type 2 diabetes [18].

Thus, the inverse and graded association between serum TB levels and arterial stiffness in this study provides significant insight into the role of serum TB levels in diabetic vascular disease as it does in the general population. In accordance with our data, certain cross-sectional studies have reported that serum bilirubin levels are inversely associated with diabetic microvascular complications, including retinopathy [19], nephropathy [20], and cardiovascular autonomic neuropathy [21]. Furthermore, a few prospective studies have demonstrated that serum TB levels are an independent predictor of the development of T2DM [22], diabetic nephropathy [23], and lower limb amputation [24].

### Pathogenesis

The biological mechanisms that link low serum bilirubin levels and arterial stiffness remain unclear, but there are several potential explanations. First, low bilirubin levels might be linked to arterial stiffness via a close association with CV risk factors: low serum bilirubin levels have been shown to be negatively correlated with hypertension, obesity, metabolic syndrome, diabetes mellitus, hyperlipidemia, and smoking status [6]. However, this is not a comprehensive explanation because the association between bilirubin levels and arterial stiffness remained significant in other studies and in ours even after adjusting for risk factors. Second, and more importantly, low bilirubin levels might be directly linked to vascular complications via decreased antioxidant defenses. Bilirubin is the end product of heme catabolism and has a powerful antioxidant capacity: as little as 10 nM TB scavenges a 10,000-fold higher concentration of hydrogen peroxide, thereby making a significant contribution to the plasma antioxidant capacity [5]. In addition, bilirubin inhibits the protein kinase C and NAD(P)H oxidase pathway that generate oxidants, uncouples eNOS, leads to endothelial dysfunction [25], and suppresses the peroxidation of lipid and lipoproteins, a crucial step in the initiation and progression of atherosclerosis [26]. Third, the anti-inflammatory properties of bilirubin could explain the arterial stiffness in patients with low bilirubin levels. Experimental studies have demonstrated that bilirubin interferes with the expression of cell adhesion molecules, complement activity, and T cell differentiation [6]. Furthermore, clinical studies have reported an inverse relationship between the levels of bilirubin and CRP, a robust marker of inflammatory status [27]. Fourth, the cardioprotective effects of bilirubin could be related to concomitant alterations in the activity of enzymes involved in the bilirubin metabolism pathway. Increased activity of heme oxygenase-1 (HMOX1), which generates CO and biliverdin (subsequently converted to bilirubin), has been reported to exert anti-atherogenic properties and promote vascular repair [28], and HMOX gene promoter polymorphism has been linked to the susceptibility to CAD in diabetic patients through its influence on serum bilirubin and ferritin levels [29]. In addition, low bilirubin might confer a heritable CVD risk via uridine diphosphate glycosyltransferase 1 (UGT1A1) genetic variants by regulating the serum bilirubin concentration. However, the evidence for the association between genetic variations of UGT1A1 and CVD risk is inconclusive [6], whereas serum bilirubin levels have been consistently shown to be inversely related to CVD.

### Gender differences

Interestingly, the impact of low bilirubin levels on arterial stiffness was apparent in the multivariate analysis in women but not in men. Research is inconclusive whether gender differences exist in the association of TB with the risk of CVD. Several studies found a marked relationship in men between bilirubin levels and a lower risk of CVD but a less clear relationship in women [30]–[34]. Some authors suggested that estrogen may be responsible for sex disparity, because it could influence on both bilirubin excretion by induction of UDP-glucuronyltransferase and PWV by improving arterial compliance [35]. On the contrary, other studies reported an inverse relationship between bilirubin and the risk of CVD equally in both sexes [9], [36]. Moreover, researchers have reported inconsistent results with regard to the association of bilirubin levels and the risk of CVD in men and women stratified by hypertension, raising the possibility that the association could be confounded by lifestyle factors and presence of disease states other than estrogen levels [37]. The discrepancy between the current results and those of prior studies may have resulted from differences in the population characteristics; the current study was performed only on patients with diabetes. Considering that women were shown to be less susceptible to low levels of bilirubin than men due to biology or to lifestyle factors in a previous study, we speculate that the potential protection offered by higher bilirubin levels might be apparent in subjects with diabetes. Diabetes tends to eliminate the "female advantage" and render the patients more vulnerable to oxidative damage. However, we cannot explain the lack of association between TB and PWV in diabetic men. Moreover, we cannot conclude that the association truly differs by sex because there was a huge difference between men and women in baseline clinical characteristics including lifestyle factors, triglycerides, high-density lipoprotein cholesterol levels and 10-year FRS, which could override any potential benefit associated with higher bilirubin levels. Further studies are warranted to explore and confirm this gender-based divergence.

### Clinical implications

In accordance with previous data on a general population, our findings suggested that a low bilirubin concentration is a simple, inexpensive, and readily available biomarker of cardiovascular risk and poor prognosis in type 2 diabetic women. Longitudinal studies are necessary to determine whether serum bilirubin levels have predictive value for CVD in women with diabetes, and future investigational studies could ascertain whether modulating bilirubin levels could be a potent therapeutic target for preventing CVD.

### Limitations

This study had some limitations. Because of its cross-sectional nature, we could not infer any causal or temporal relationships between bilirubin levels and the coronary artery burden. Second, we measured baPWV rather than the preferred measurement of aortic stiffness. Third, only single serum TB measurements were available, which was not as desirable as using the mean of several measurements. However, the use of standardized methods in a single center and taking measurements from fasting subjects should have improved the reliability of the data because there was less fluctuation in TB levels than in the postprandial state.

## Conclusion

Low TB levels were significantly associated with arterial stiffness in Korean women with type 2 diabetes. Our data suggested that bilirubin may protect against macrovascular disease in women with type 2 diabetes, similar to its protective effects in the general population.
